# **Sigmoid volvulus in pregnancy and puerperium: a surgical and obstetric catastrophe. Report of a case and review of the world literature**

**DOI:** 10.1186/1749-7922-7-10

**Published:** 2012-05-02

**Authors:** Muhammad R Khan, Sameer ur Rehman

**Affiliations:** 1 Department of Surgery and Medical College, Aga Khan University & Hospital, Stadium Road, Karachi, 74800, Pakistan

**Keywords:** Sigmoid volvulus, Pregnancy, Outcome

## Abstract

Sigmoid volvulus is a rare surgical complication occurring in pregnancy and puerperium. Only 84 cases of sigmoid volvulus in pregnancy have been reported in the English literature so far. We have reviewed the available literature on this subject and present another case recently managed at our institution. The available literature suggests that over the years, there has been an improvement in the maternal and fetal outcome for this critical condition, but delay in presentation and a further delay in diagnosis remain a challenge for the treating physicians. Our patient was a 30-week pregnant lady, who presented late with 6 days history of abdominal pain, distension and absolute constipation. She had evidence of multi-organ dysfunction at presentation due to complicated sigmoid volvulus. She was resuscitated and surgical exploration revealed gangrenous large bowel. Bowel resection with diverting ileostomy was performed, but she succumbed to the septic shock due to late presentation. Acute surgical pathology may be overlooked in pregnant patients due to reluctance in radiological workup and a high index of suspicion is essential for enhanced outcome. There is a need to increase the awareness amongst the obstetricians and general practitioners. Early diagnosis and referral and timely surgical intervention could significantly improve the outcome of this surgical and obstetric catastrophe.

## Background

Sigmoid volvulus in pregnancy is a rare but serious complication associated with a significant maternal and fetal mortality [1]. The fundamental problem of sigmoid volvulus in pregnancy is that of delay in presentation and further delay in diagnosis leading to ischemia of the colon, which requires bowel resection and colostomy as seen in most of the reported cases [2-20]. Timely surgical intervention is essential to reduce maternal and fetal morbidity and mortality [1]. Perforation, peritonitis and sepsis can be the maternal complications if intervention is not done early in the course of the disease. The fetal complications include preterm delivery, intrauterine death and neonatal sepsis. We have reviewed the available literature on this subject and report another case of a 30-week pregnant lady who presented to us with complicated sigmoid volvulus (Table 1). There is a need to increase the awareness amongst the general practitioners and community obstetricians for this potentially life threatening condition. A high index of suspicion and judicious use of modern radiological imaging may help make an early diagnosis and improve the maternal and fetal outcomes.

**Table 1:** Reported Cases of Sigmoid Volvulus in Pregnancy and Puerperium

Authors	Year of the study	Number of cases reported	Stage of pregnancy in weeks	Duration of symptoms in days	Status of colon	Treatment offered	Outcome	
							Mother	Fetus
Recent Cases	1978 – 2011	20						
Present case	2011	1	30	6 days	Gangrenous	Total colectomy with proximal ileostomy	Expired	IUD
Kolusari A et al [2]	2009	4	7	1	Gangrenous	Resection of sigmoid colon with primary anastomosis	Healthy	Alive
			31	2	Gangrenous	Resection of sigmoid colon with proximal colostomy	Healthy	IUD
			32	2	Gangrenous	Resection of sigmoid colon with proximal colostomy	Healthy	Alive
			Puerperium	3	Gangrenous	Resection of sigmoid colon with proximal colostomy	Expired	Alive
Vo TM et al [3]	2008	1	28	1	Gangrenous	Resection of sigmoid colon with proximal colostomy	Healthy	Alive
Iwamoto I et al [4]	2007	1	35	3	Perforated	Repair of perforation with proximal colostomy	Expired	IUD
Sascha Dua R et al [5]	2007	1	Puerperium	1	Viable	Laparotomy and sigmoidopexy	Healthy	Alive
Machado NO et al [6]	2006	1	18	1	Viable	Resection of sigmoid colon with primary anastomosis	Healthy	Alive
Alshawi JS [7]	2005	1	28 & 35 (recurrent)	1	Viable	Sigmoidoscopy and decompression followed by elective sigmoid colectomy	Healthy	Alive
De U et al [8]	2005	1	24	3	Gangrenous	Resection of sigmoid colon with proximal colostomy	Healthy	IUD
Joshi MA et al [9]	1999	1	28	2	Gangrenous	Resection of sigmoid colon with proximal colostomy	Healthy	IUD
Lurie S et al [10]	1997	1	Ectopic Pregnancy	2	Viable	Laparotomy and de-torsion of volvulus	Healthy	Ectopic
Lord SA et al [11]	1996	1	36	2	Gangrenous	Resection of sigmoid colon with proximal colostomy	Healthy	Alive
Allen JC [12]	1990	1	28	1	Viable	Sigmoidoscopy and decompression	Healthy	Alive
Keating JP et al [13]	1985	1	34	1	Viable	Resection of sigmoid colon with double barrel colostomy	Healthy	Alive
Hofmeyr GJ et al [14]	1985	2	33	3	Gangrenous	Resection of sigmoid colon with proximal colostomy	Healthy	IUD
			26	3	Gangrenous	Resection of sigmoid colon with proximal colostomy	Expired	IUD
Fraser JL et al [15]	1983	1	32	1	Viable	Laparotomy and de-torsion of volvulus	Healthy	Alive
Fuller JK et al [16]	1978	1	Puerperium	1	Viable	Laparotomy and de-torsion of volvulus	Healthy	Alive
Lazaro EJ et al [17]	1958 – 1969	13						
Harer WB Jr. et al [18]	1944 – 1958	11						
Kohn SG et al [19]	1931 – 1944	12						
Lambert AC [20]	Before 1931	29						

Legend: IUD = Intrauterine Death

## Case presentation

A 25-year-old pregnant lady, gravida 2 and para 1, presented to the emergency room with 6 days history of lower abdominal pain, abdominal distension, bilious vomiting and constipation. She had no significant past medical or surgical history and her menstrual and antenatal history were uneventful. She was being managed conservatively for these complaints at a local hospital before presenting to us in the emergency. On examination, she was dehydrated, tachycardiac with a heart rate of 136 beats per minute and tachypneic with a respiratory rate of 28 per minute. Her initial blood pressure was 102/69 mmHg and she was drowsy. Her abdomen was distended and tense, with generalized tenderness and a tympanitic note on percussion. The uterine size was of 30 weeks gestation. Rectal examination revealed no stool and cervical os was closed on per vaginal examination. She was clinically suspected to have peritonitis with evidence of septic shock. She was resuscitated with intravenous fluids and antibiotics.

Plain radiographs of the chest and abdomen showed pneumoperitoneum, a single fetus and a distended gas filled transverse colon. Ultrasound scan of the abdomen revealed moderate amount of free fluid with mobile internal echoes representing bowel content and intra-uterine death of the fetus. Her laboratory workup revealed severe electrolyte imbalance with serum potassium of 6.3 mmol/L (normal 3.5-5) and blood glucose level of 39 mg/dL (normal 80–160). She had evidence of severe metabolic acidosis with serum pH of 7.18 (normal 7.36-7.44), hypoxia with pO2 of 39 mmHg (normal 85–105) and deranged coagulation. The surgical and obstetric teams in the emergency room evaluated the patient. While being resuscitated in the emergency room, the conscious level of the patient dropped further and she was intubated and put on the mechanical ventilator.

With the clinical diagnosis of bowel perforation and peritonitis, the patient was taken up for emergency laparotomy. Intra-operatively findings were of sigmoid volvulus resulting in closed loop obstruction leading to distension and ischemia of whole large bowel. The whole of the colon was dilated, friable, and gangrenous. Multiple perforations were identified in the colon with around 800 ml of feculent material aspirated on opening the abdomen. Whole colon was mobilized & resected and diverting ileostomy with a Hartman’s procedure was done. A lower segment caesarean section was done for delivering the dead fetus and modified B-lynch sutures applied to the uterus. Post-operatively, she was continued on broad-spectrum antibiotics and shifted to the intensive care unit. She had an initial period of recovery for a couple of days, but subsequently, her pulmonary function deteriorated with development of pneumonia and adult respiratory distress syndrome. In addition to high ventilator support, she also needed increasing dose of inotropes and eventually expired on the 8^th^ post-operative day due to overwhelming sepsis and organ dysfunction.

## Discussion

The incidence of intestinal obstruction in pregnancy ranges from 1 in 1500 to 1 in 66431 deliveries [2]. Intestinal obstruction in pregnancy can be caused by many factors including congenital or postoperative adhesions, volvulus, intussusceptions, hernia and appendicitis [1]. Sigmoid volvulus is the most common cause of bowel obstruction complicating pregnancy, accounting for up to 44 per cent of cases [21]. Pregnancy itself is considered to be the precipitating factor for sigmoid volvulus. The occurrence of sigmoid volvulus in pregnancy is due to displacement, compression and partial obstruction of a redundant or abnormally elongated sigmoid colon by the gravid uterus [18]. This could probably explain the increased incidence of sigmoid volvulus in the third trimester of pregnancy [1]. Despite this higher propensity in the third trimester, there have been reports of this complication developing in the early pregnancy as well as the puerperium [2,5,16,18].

To date, 84 cases of sigmoid volvulus have been reported occurring in the pregnancy and puerperium (Table 1). Lambert [20] reported 29 cases of sigmoid volvulus before 1931, followed by another 12 cases reported by Kohn et al [19] between 1931 and 1944. Subsequently, all the previously reported cases were reviewed by Harer et al [18] in 1958, who reported an additional 11 cases between 1994 and 1958. Later on, Lazaro et al [17] compiled another 13 cases occurring between 1558 and 1969. In this report, we have identified 19 more cases reported till 2009, and include another case managed recently at our institution.

The diagnosis of sigmoid volvulus is suspected when a pregnant female presents with a clinical triad of abdominal pain, distention, and absolute constipation. The average time from the onset of obstructive symptoms until presentation has been reported to be 48 hours [1]. This is largely because pregnancy itself masks the clinical picture since abdominal pain, nausea, and leukocytosis can occur in an otherwise normal course of pregnancy [13]. In our review of recent 20 cases, the mean delay between the onset of symptoms to presentation was 2 days, with a range from few hours to as many as 6 days, as seen in our case. Six patients presented more than 48 hours after the onset of symptoms. Harer et al [18] also noted similar delay in presentation in their review and concluded that such a delay in diagnosis and surgical intervention had a significant impact on the ultimate outcome of the mother and fetus.

The maternal and fetal outcome in sigmoid volvulus has been directly related to the degree of bowel ischemia and subsequent systemic sepsis. In our analysis of recent 20 cases, there were 4 (20 %) maternal and 8 (40 %) fetal deaths, including one ectopic pregnancy. It is important to note that all the maternal deaths occurred in the group of patients where delay in presentation and surgical intervention was more than 2 days. [2,4,14] Similarly, 5 fetal deaths were seen in patients who presented after 48 hours of onset of symptoms, as compared to 2 fetal deaths in patients presenting early in the course of the disease. This observation highlights the fact that high index of clinical suspicion is vital in cases of intestinal obstruction in pregnant patients. This facts needs to be emphasized amongst the general practitioners and community obstetricians primarily responsible for taking care of these patients.

Another important area of concern is the reluctance in the utilization of modern radiological diagnostic tools in pregnant patients. There have always been concerns about the radiation exposure of the fetus during pregnancy. Significant radiation exposure may lead to chromosomal mutations, neurologic abnormalities, mental retardation, and increased risk of childhood leukemia. Cumulating radiation dosage is the primary risk factor for adverse fetal effects, but fetal age at exposure is also important [22-24]. Exposure during the first week of gestation results in highest rates of fetal mortality. The next most sensitive time period is between 10 and 17 weeks of gestation, when central nervous system teratogenesis becomes an important consideration. After this period, the concern shifts from teratogenesis to the risk of childhood hematologic malignancy. It has been recommended that the cumulative radiation dose to the fetus during pregnancy should be less than 5–10 rads [25]. In general, no single diagnostic study exceeds 5 rads of radiation exposure. As an example, the radiation dose to the fetus for a plain abdominal radiograph averages 0.1–0.3 rads, while a CT of the pelvis and abdomen yields up to 5 rads of fetal exposure [26]. In any case, the health and life of the mother takes priority over the concerns for the fetus and judicious use of radiation may help make an early diagnosis with optimal outcome for both the mother and the fetus.

The management of intestinal obstruction and perforation in pregnant women is pretty much similar to that of non-pregnant women. The basis of therapy is early surgical intervention [27]. Surgery should be performed via midline vertical laparotomy. In the third trimester, if sufficient intestinal exposure cannot be obtained due to enlarged uterus, a caesarean section must be carried out [28]. The entire bowel should be examined for other areas of obstruction. Intestinal viability should be assessed cautiously and segmental resection with or without anastomosis is often necessary [27].

## Conclusions

Sigmoid volvulus complicating pregnancy is very rare condition with significant maternal and fetal morbidity and mortality. Timely diagnosis mandates high index of clinical suspicion in patients presenting with abdominal pain, distension and absolute constipation. Hesitancy in getting X-rays in view of pregnant situation must be avoided and appropriate management must be defined. Delay in diagnosis and treatment beyond 48 hours results in increased fetal and maternal morbidity and mortality. Review of the available literature emphasizes the importance of early diagnosis and timely intervention to minimize maternal and fetal morbidity and mortality.

## Consent

A written informed consent was obtained from the next of kin of the patient for publication of this Case report and any accompanying images. A copy of the written consent is available for review by the Editor-in-Chief of this journal.

## Competing interests

The author declares that they have no competing interest.

## Authors’ contribution

MRK conceived the study. SR collected the data and prepared the initial manuscript. The manuscript was critically reviewed and approved by all the authors.
